# Orthogonal Band Planning and Synergistic Interference Suppression for Full-Duplex Acoustic Telemetry in Coiled Tubing of Deep Horizontal Wells

**DOI:** 10.3390/s26123929

**Published:** 2026-06-20

**Authors:** Hao Geng, Yingjian Xie, Junlong Wu, Zhihao Wang, Hu Han, Dong Yang

**Affiliations:** 1 Hubei Key Laboratory of Oil and Gas Drilling and Production Engineering, Yangtze University, Wuhan 430100, China; 2022710259@yangtzeu.edu.cn (H.G.); 2025720454@yangtzeu.edu.cn (Z.W.); hanhu@yangtzeu.edu.cn (H.H.); 2 School of Petroleum Engineering, Yangtze University, Wuhan 430100, China; 3 State Key Laboratory of Low Carbon Catalysis and Carbon Dioxide Utilization, Yangtze University, Wuhan 430100, China; 4 China National Petroleum Offshore Engineering Co., Ltd., Drilling Branch, Tianjin 300467, China; wjl18389117787@163.com

**Keywords:** coiled tubing, full-duplex acoustic telemetry, dispersion characteristics, orthogonal frequency-band planning, synergistic interference suppression

## Abstract

**Highlights:**

**What are the main findings?**

- An acoustic sensing and telemetry framework was established by coupling waveguide modeling, orthogonal frequency-band planning, and adaptive interference suppression.
- Full-scale tests demonstrated reliable full-duplex transmission over a 457.2 m coiled-tubing loop under turbulent flow rates up to 400 L/min.

**What are the implications of the main findings?**

- The study demonstrates that sensor-level mechanical decoupling, frequency-domain isolation, and CEEMDAN–wavelet denoising can be integrated to improve acoustic telemetry robustness.
- The proposed method supports real-time bidirectional downhole measurement and control without relying on wired coiled-tubing transmission.

**Abstract:**

Full-duplex acoustic telemetry is important for real-time bidirectional measurement and control in intelligent coiled-tubing operations, but its reliability in deep horizontal wells is limited by long-range dispersion, asymmetric flow-induced noise, and severe near-end self-interference. This study proposes an orthogonal frequency-band planning and synergistic interference suppression method for full-duplex acoustic communication in coiled tubing. A dispersion model and an asymmetric attenuation model were first established for a fluid-filled coiled-tubing cylindrical-shell waveguide to characterize the physical transmission constraints. A multiphysics multi-objective cost function was then formulated by considering dispersion flatness, channel attenuation, asymmetric noise adaptability, and spectral isolation, and an improved simulated annealing algorithm was used to optimize the uplink and downlink frequency bands. In addition, a three-stage suppression architecture integrating mechanical decoupling, physical-layer frequency isolation, and CEEMDAN–wavelet denoising was developed to reduce self-interference and residual nonstationary noise. Full-scale experiments on a 457.2 m coiled-tubing surface circulation system showed that the proposed method improved the output signal-to-interference-plus-noise ratio from −15 dB to 18.5 dB and maintained a bit error rate below 1.2 × 10^−4^ at 400 L/min. These results indicate that the proposed approach can enhance the robustness of full-duplex acoustic telemetry under strong flow-induced noise.

## 1. Introduction

With the advancement of unconventional oil and gas development, coiled tubing (CT) technology is critical for logging, well intervention, and intelligent drilling in deep horizontal wells [1,2]. In complex downhole environments, real-time, high-speed bidirectional data exchange between bottomhole tools and surface control systems is a prerequisite for improving the efficiency and safety of closed-loop operations [3]. Recently, overcoming extreme attenuation and nonstationary noise in harsh acoustic and fluid channels has become a prominent research focus. Latest studies have proposed various advanced techniques, ranging from machine learning-driven underwater covert communication architectures to continuous-phase chirp spread spectrum for mud-pulse telemetry, aiming to break the physical bandwidth barriers [4,5]. However, conventional mud-pulse telemetry provides low data rates and is constrained by the compressibility of multiphase fluids [6,7,8]. Electromagnetic telemetry, by contrast, suffers from signal attenuation that is highly sensitive to formation resistivity, which severely limits its transmission depth [9,10,11]. Wired coiled tubing (WCT) provides high-bandwidth transmission, yet its internal cable occupies significant flow area, causing severe pressure drops and limiting the maximum pumping rate [12,13]. In addition, under repeated spooling and harsh fluid-circulation conditions, the internal cable and its connectors are highly susceptible to mechanical fatigue and chemical corrosion [14,15]. In comparison, acoustic telemetry uses the coiled-tubing string or the internal fluid as the physical transmission channel, offering significant advantages such as wide bandwidth, high transmission rate, and immunity to formation electromagnetic properties. It has therefore emerged as one of the most promising alternative communication technologies for extreme deep-subsurface exploration environments [16,17,18].

Despite its considerable potential, establishing a highly reliable bidirectional acoustic data link during high-flow-rate circulation operations in coiled tubing still faces multiple physically destructive challenges [19,20]. First, full-duplex communication inevitably introduces strong near-end self-interference, which severely masks the weak signals received from the far end [21,22]. In the analogous domain of underwater acoustic communications, comprehensive literature reviews have demonstrated that sophisticated signal processing paradigms ranging from standard adaptive digital SIC to advanced biologically inspired noise adaptation can effectively mitigate severe multipath and environmental interference [23,24]. To break the half-duplex throughput limitation, in-band full-duplex acoustic communications and sophisticated spatial–digital self-interference cancelation mechanisms have drawn significant attention over the past two years. Recent breakthroughs highlight the necessity of combining hardware isolation with adaptive filtering to mitigate near-end strong leakage [25]. While these extensive underwater acoustic methodologies provide a foundational reference for self-interference cancelation, their direct application in coiled tubing is heavily constrained [26]. Second, the downhole fluid environment is extremely harsh [27]. In particular, under high mud-pump flow rates, high-velocity fluid impingement on the tubing wall and throttling components generates intense, high-energy, and nonstationary turbulent noise [28,29]. In addition, the inherent dispersion and attenuation of acoustic waves during long-distance propagation along the tubing string further cause severe waveform distortion and a rapid degradation in the signal-to-noise ratio (SNR) [30,31,32].

At present, research on noise suppression for acoustic telemetry is largely confined to backend digital signal processing, such as conventional bandpass filtering, adaptive filtering, and standard wavelet denoising methods [33,34]. Furthermore, hybrid algorithms integrating CEEMDAN with advanced wavelet thresholding have recently been proven to be highly robust for separating nonstationary multimodal acoustic signals in complex industrial environments [35]. These state-of-the-art developments provide a solid methodological inspiration for isolating target signals from dynamic fluid noise in coiled tubing. However, these approaches generally rely on the idealized white-noise assumption and therefore have limited capability in effectively separating strong turbulent scattering noise that spectrally overlaps with the useful signal [36,37]. Furthermore, although emerging deep learning-based techniques have demonstrated robust noise suppression capabilities in surface systems, their practical deployment in real-time downhole acoustic telemetry is severely hindered by the stringent power limits, inherent computational constraints, and critical latency requirements of embedded logging tools. More critically, existing studies tend to treat “frequency-band allocation” and “signal extraction” as isolated problems, lacking a globally coordinated framework spanning from physical-layer channel characteristics to digital-layer signal processing [38]. For full-duplex systems, once the initial orthogonal frequency-band planning is improperly designed, merely relying on backend digital filtering can easily lead to the complete collapse of the communication link [39].

To address these bottlenecks, this paper proposes a multiphysics framework for orthogonal frequency-band planning and synergistic interference suppression in full-duplex acoustic telemetry. The main contributions of this work are as follows.

(1) By jointly considering the physical boundaries of the transducers, dispersion, attenuation, and asymmetric pumping noise, a multi-objective coupled cost function is established, and an improved simulated annealing (ISA) algorithm is introduced for global optimization, thereby enabling optimal orthogonal frequency-band allocation for noise avoidance at the physical layer.
(2) To suppress residual in-band nonstationary fluid noise and near-end self-interference, a CEEMDAN–wavelet joint denoising framework based on cross-correlation adaptive decision is proposed, enabling accurate separation of mixed-modal characteristic signals.
(3) Relying on a full-scale coiled-tubing surface circulation experimental system, comprehensive tests are conducted under extreme turbulent flow rates ranging from 0 to 400 L/min, verifying the reliable robustness of the proposed integrated framework in maintaining reliable communication at the forward-error-correction limit.

To ensure the statistical rigor and reproducibility of the proposed framework, the experimental evaluation incorporates multiple repeated trials to determine standard deviations and confidence boundaries. Furthermore, a comprehensive sensitivity analysis is conducted to evaluate the stability of the optimized frequency allocation and joint denoising parameters under volatile downhole dynamics, establishing a robust benchmarking baseline against conventional adaptive signal cancelation methodologies.

## 2. Materials and Methods

This section systematically presents the theoretical foundations, algorithm design, hardware architecture, and experimental procedures involved in this study, with the aim of fully clarifying the design rationale and implementation pathway of the full-duplex acoustic communication system for coiled tubing and ensuring the reproducibility and verifiability of the research process. First, a comprehensive full-duplex channel model for fluid-filled coiled tubing is established based on elastodynamic theory to reveal the propagation behavior and physical constraint boundaries of acoustic waves in a cylindrical shell waveguide. On this basis, a multi-objective optimization model for full-duplex frequency-band planning is constructed, and an improved simulated annealing algorithm is designed to achieve global optimization of orthogonal frequency bands. Subsequently, a three-stage synergistic interference suppression architecture integrating mechanical, physical-layer, and digital-layer techniques is proposed to address the near-end self-interference problem in full-duplex concurrent transmission. Finally, the configuration of the full-scale surface test system and the standardized testing procedures are described in detail, providing a complete methodological foundation for the subsequent performance evaluation.

### 2.1. Theoretical Model of the Full-Duplex Channel in Fluid-Filled Coiled Tubing

In downhole acoustic telemetry systems, coiled tubing constitutes a cylindrical shell waveguide filled with viscous fluid. Unlike conventional jointed drill pipes with tool joints, coiled tubing exhibits geometric continuity, and the acoustic propagation characteristics over long transmission distances are significantly constrained by dispersion effects and attenuation in non-Newtonian fluids. In this subsection, a comprehensive channel model incorporating dispersion characteristics, asymmetric attenuation, and the full-duplex self-interference mechanism is established to provide a theoretical basis for the subsequent design of frequency-band planning and interference suppression.

#### 2.1.1. Dispersion Characteristic Equation of the Cylindrical Shell Waveguide

The coiled tubing is modeled as an isotropic, infinitely long elastic cylindrical shell immersed in the borehole annular fluid and internally filled with drilling fluid. A cylindrical coordinate system is established, in which the z-axis extends along the axial centerline of the tubing, while the r,θ planes are perpendicular to the tubing axis. According to the theory of linear elastodynamic wave propagation, the displacement potential function within the tubing wall in the spatiotemporal domain can be expressed in terms of separable solutions to the Helmholtz equation [40]. For harmonic waves propagating in the axial direction, the scalar potential function is written as:

(1) $$\Phi \left(r,\theta ,z,t\right)=\left[A_{n}J_{n}\left(\lambda r\right)+B_{n}Y_{n}\left(\lambda r\right)\cos(n\theta )e^{i\left(kz-\omega t\right)}\right]$$

where r and z are the radial and axial coordinates, respectively, in m; t is the time variable, in s; Φ is the displacement potential function, in m^2^; ω is the angular frequency, in rad/s; k is the axial wavenumber, in rad/m; n is the circumferential mode order, dimensionless; λ is the radial wavenumber parameter, in rad/m; $J_{n}$ and $Y_{n}$ are the Bessel functions of the first and second kinds, respectively.

By incorporating the boundary conditions at the inner and outer interfaces of the tubing wall, the coefficient matrix D is constructed. Setting the determinant of this matrix to zero yields the dispersion characteristic equation for multilayer coupled media:

(2) $$\left|D\left(\omega ,k\right)\right|=0$$

By numerically solving the above equation, the corresponding wavenumbers for different propagation modes can be obtained. In full-duplex communication, the lowest-order longitudinal mode $L\left(0,1\right)$ is selected as the primary carrier mode to minimize dispersion during long-distance transmission. The group velocity, defined as the propagation velocity of wave-packet energy, is expressed as:

(3) $$C_{g}\left(\omega \right)=\frac{d\omega }{dk}$$

The group velocity of the $L\left(0,1\right)$ mode exhibits strong nonlinear frequency dependence near the cutoff frequency, which constitutes the fundamental physical origin of waveform distortion and intersymbol interference in long-distance broadband transmission.

#### 2.1.2. Asymmetric Attenuation Model of the Uplink and Downlink Channels

Although the acoustic waveguide itself is reciprocal, the fluid environment and noise distribution in deep-well operations render the uplink and downlink channels significantly asymmetric [41]. The total attenuation during signal transmission consists of two components and can be expressed as:

(4) $$\alpha_{\mathrm{total}}\left(f\right)=\alpha_{\mathrm{steel}}\left(f\right)+\alpha_{\mathrm{fluid}}\left(f\right)$$

where $\alpha_{\mathrm{steel}}$ is the attenuation caused by internal material damping of the tubing wall, which is usually linearly related to frequency, and $\alpha_{\mathrm{fluid}}$ is the coupled attenuation induced by the viscous resistance of the drilling fluid inside the tubing.

In deep-well operations, drilling fluids are commonly non-Newtonian, and the shear damping they impose on acoustic waves increases quadratically with frequency. Based on the modified Stokes–Kirchhoff formulation, the attenuation coefficient can be expressed as:

(5) $$\alpha_{\mathrm{fluid}}\left(f\right)=\frac{1}{d}\sqrt{\frac{\pi f\eta }{\rho_{f}c_{f}^{2}}}$$

where f is the signal frequency, in Hz; d is the inner diameter of the coiled tubing, in m; η is the dynamic viscosity of the fluid, in Pa·s; $\rho_{f}$ is the fluid density, in kg/m^3^; $c_{f}$ is the sound velocity in the fluid, in m/s.

It can be seen from the above expression that the fluid-induced attenuation is proportional to the square root of frequency. To explicitly account for the rheological nonlinearities inherent in actual drilling muds exhibiting Bingham or Herschel–Bulkley behaviors, the dynamic viscosity parameter in this formulation represents an effective apparent viscosity. This apparent viscosity is dynamically evaluated based on the localized shear rate of the non-Newtonian fluid under specific pumping profiles. It should be noted that in the current full-scale surface experimental validation, water was utilized as the baseline Newtonian fluid. This controlled approach rigorously isolates the kinematic effects of high-velocity turbulent flow on the acoustic channel, thereby establishing a fundamental physical benchmark before introducing the compounding variables of complex slurry rheology in future field trials. Meanwhile, the channel background noise field exhibits strong spatial asymmetry: the receiver in the downlink channel is dominated by broadband drill-string vibration noise, whereas the receiver in the uplink channel is dominated by low-frequency, high-energy pumping noise.

**Figure 1:**
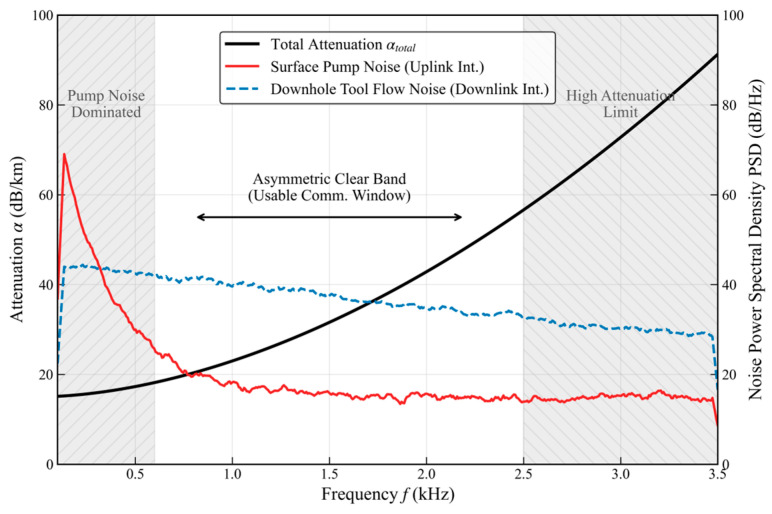
Joint analysis of the asymmetric noise field and attenuation-limited physical model in the coiled tubing acoustic channel.

To quantitatively characterize the physical constraint boundaries encountered by full-duplex communication over long-distance coiled tubing, the above attenuation model is jointly analyzed together with the power spectral densities of the uplink and downlink background noise. As shown in Figure 1, the channel environment exhibits pronounced frequency-domain asymmetry and boundary-constrained characteristics.

The following physical mechanisms can be directly observed from Figure 1. First, in the low-frequency band, although the acoustic attenuation introduced by the channel itself, $\alpha_{\mathrm{total}}$, is relatively small, the fluid pulsation noise generated by the reciprocating motion of the surface high-pressure plunger pump exhibits extremely large energy peaks. This intense low-frequency noise field directly overwhelms the uplink receiver, forming a “low-frequency noise wall.” Second, in the high-frequency band, as the frequency increases, acoustic energy dissipation caused by fluid viscosity and radial displacement of the tubing wall rises sharply in a nonlinear manner, giving rise to a high-attenuation constrained region in which weak signals can hardly be sustained. Finally, it is worth noting that the downlink receiver located at the bottom hole is mainly affected by the hydrodynamic noise generated by downhole tools. The turbulent noise produced when high-pressure fluid passes through restricted flow passages exhibits a broadband distribution in the frequency domain, and its overall energy level is much lower than that of the surface pumping noise. Based on the above analysis, it can be concluded that, because the uplink and downlink are subject to fundamentally different noise-boundary constraints, the conventional symmetric frequency-band partitioning strategy is no longer applicable under such operating conditions. Instead, the system must exploit the asymmetry of the channel parameters to precisely optimize and occupy the “asymmetric clear window” indicated in the figure, located between the low-frequency pump-noise-dominated region and the high-frequency attenuation-constrained region. This physical insight also provides the core theoretical basis for the subsequent construction of a nonconvex multi-objective cost function and the implementation of synergistic interference suppression.

#### 2.1.3. Full-Duplex Self-Interference and Signal-to-Interference-Plus-Noise Ratio Model

In the single-tube full-duplex mode, the high-power signal transmitted by the local transmitter is directly coupled to the high-sensitivity receiver through the tubing wall, thereby generating near-end self-interference [42]. The mixed signal acquired by the receiving sensor can be expressed as:

(6) $$y\left(t\right)=s_{\mathrm{leak}}\left(t\right)+s_{\mathrm{remote}}\left(t\right)+n\left(t\right)$$

where $s_{\mathrm{leak}}\left(t\right)$ is the local leakage signal; $s_{\mathrm{remote}}\left(t\right)$ is the desired signal after long-distance transmission; $n\left(t\right)$ is the environmental noise. To quantify the interference rejection capability of the system, the signal-to-interference-plus-noise ratio (SINR) in the frequency domain is defined as:

(7) $$\mathrm{SINR}\left(f\right)=10{log}_{10}\left(\frac{P_{\mathrm{rx}}\left(f\right)}{P_{\mathrm{leak}}\left(f\right)\cdot A_{\mathrm{iso}}\left(f\right)+N_{\mathrm{floor}}\left(f\right)}\right)$$

where $P_{\mathrm{rx}}\left(f\right)$ is the power spectral density of the remotely received signal, in V^2^/Hz; $P_{\mathrm{leak}}\left(f\right)$ is the power spectral density of the local leakage signal, in V^2^/Hz; $A_{\mathrm{iso}}\left(f\right)$ is the isolation attenuation factor provided by physical frequency-band planning, dimensionless; $N_{\mathrm{floor}}\left(f\right)$ is the environmental noise floor, in V^2^/Hz. It is worth noting that the unit V2/Hz applied for the power spectral density parameters intrinsically assumes a normalized reference load impedance of 1 Ω. This formulation follows standard digital signal processing conventions, enabling the signal power to be mathematically represented directly by the square of the voltage amplitude without requiring specific transducer impedance calibrations in the algorithmic derivation.

This model indicates that minimizing the spectral overlap between the local leakage signal and the remotely received signal through orthogonal frequency-band planning is the physical foundation for improving the signal-to-interference-plus-noise ratio of the full-duplex system.

### 2.2. Multi-Objective Optimization Model for Full-Duplex Orthogonal Frequency-Band Planning

The frequency-band selection of a full-duplex acoustic communication system for coiled tubing involves stringent physical tradeoffs. Low-frequency bands exhibit relatively low attenuation but suffer from severe pumping-noise interference, whereas high-frequency bands have lower background noise but are subject to much stronger signal attenuation and dispersion effects [43]. Meanwhile, the full-duplex mode requires the uplink and downlink channels to remain highly orthogonal in the frequency domain in order to suppress self-interference. In essence, this problem is a multi-objective resource allocation problem under nonconvex physical constraints. In this subsection, the optimization variables and constraint space are explicitly defined, and a multiphysics-coupled cost function is constructed to quantitatively evaluate the overall performance of candidate frequency-band allocation schemes.

#### 2.2.1. Definition of Optimization Variables and Constraint Space

To achieve fine-grained allocation of spectral resources, the uplink carrier center frequency, downlink carrier center frequency, and effective bandwidth of a single channel are defined as the optimization parameter vector [44], which can be expressed as:

(8) $$x={\left[f_{u},f_{d},B\right]}^{T}$$

where x is the frequency-band parameter vector to be optimized; $f_{u}$ is the center frequency of the uplink communication; $f_{d}$ is the center frequency of the downlink communication; B is the bandwidth of the unidirectional communication signal, in Hz.

The solution space of the parameter vector is subject to hard constraints imposed by the physical devices and channel characteristics. The constraints are given by:

(9) $$s.t.\left\{\begin{array}{l} f_{\min}\leq f_{d},f_{u}\leq f_{\max}\\ \left|f_{u}-f_{d}\right|\geq B+G_{\min}\\ B\geq R_{b}/\eta_{\mathrm{spec}} \end{array}\right.$$

where $f_{\min}$ and $f_{\max}$ are the lower and upper cutoff frequencies of the linear response range of the transducer, respectively, in Hz; $G_{\min}$ is the minimum guard band required by the physical-layer filter, in Hz; $R_{b}$ is the target communication rate, in bps; $\eta_{\mathrm{spec}}$ is the spectral efficiency of the modulation/demodulation scheme, in bps/Hz.

The above constraints ensure that the selected frequency bands are physically excitable by the hardware, nonoverlapping in the frequency domain, and capable of satisfying the minimum required communication rate.

#### 2.2.2. Construction of the Multiphysics-Coupled Cost Function

To quantitatively evaluate the quality of a given frequency-band allocation vector, a normalized multi-objective cost function is established [45]. This function aims to minimize the overall cost of the frequency-band planning scheme in terms of dispersion, transmission loss, and interference, and is expressed as:

(10) $$J\left(x\right)=w_{1}J_{\mathrm{disp}}+w_{2}J_{\mathrm{att}}+w_{3}J_{\mathrm{asym}}+w_{4}$$

where $w_{1}$ to $w_{4}$ are dimensionless weighting coefficients. In this study, these parameters are systematically determined through a heuristic calibration procedure driven by empirical channel measurements. Specifically, to prioritize the mitigation of severe intersymbol interference and high-energy surface pumping noise inherent in deep-well operations, the dispersion and asymmetric noise adaptation terms are assigned primary weights of 0.3 ($w_{1}$ = 0.3, $w_{3}$ = 0.3). The attenuation and spectral isolation terms are allocated secondary weights of 0.2 ($w_{2}$ = 0.2, $w_{4}$ = 0.2) to maintain basic transmission energy and channel orthogonality. Sensitivity evaluations demonstrate that the global optimal solution exhibits strong stability, maintaining consistent convergence even when these heuristically chosen weights are subjected to ±10% random fluctuations. This robustness ensures the broad applicability and reproducibility of the method across different tubing configurations. The physical definitions of the individual sub-cost terms are given as follows.

(1) Dispersion cost term $J_{\mathrm{disp}}$

To reduce waveform distortion after long-distance transmission, the group velocity $C_{g}$ should remain as constant as possible within the selected frequency band. Accordingly, $J_{\mathrm{disp}}$ is defined as cumulative integral of the absolute rate of change in group velocity with respect to frequency, evaluated independently across both the bidirectional uplink and downlink frequency band:

(11) $$J_{disp}=\int_{f_{d}-B/2}^{f_{d}+B/2}{\left|\frac{dC_{g}}{df}\right|}^{2}df+\int_{f_{d}-B/2}^{f_{d}+B/2}{\left|\frac{dC_{g}}{df}\right|}^{2}df$$

where $C_{g}$ is the group velocity dispersion rate, in m/s^2^. This term forces the carrier frequency to avoid the strong-dispersion region near the cutoff frequency and instead preferentially select the linear plateau region of the group-velocity curve, thereby suppressing intersymbol interference.

(2) Attenuation cost term $J_{\mathrm{att}}$

To maximize the received signal energy, the selected frequency band should lie in a region where the total attenuation coefficient $a_{total}$ is relatively low [46]. Considering that the downlink usually carries high-power control commands, whereas the uplink signal is relatively weak, this term mainly imposes a penalty on the uplink channel:

(12) $$J_{att}=\frac{1}{B}\int_{f_{u}-B/2}^{f_{u}+B/2}\alpha_{total}(f)df$$

where $a_{total}$ is the total channel attenuation coefficient established in the previous subsection, in Np/m. This integral mean reflects the average signal energy loss per unit transmission distance.

(3) Asymmetric noise adaptation term $J_{\mathrm{asym}}$

This term reflects the asymmetric characteristics of the full-duplex channel. The uplink receiver is located at the surface and is dominated by low-frequency pumping noise $N_{pump}$, whereas the downlink receiver is located at the bottom hole and is mainly affected by broadband drill-string noise $N_{drill}$ [47]. To improve the signal-to-noise ratio, the uplink frequency band should be deliberately placed away from the low-frequency region where pumping noise is concentrated:

(13) $$J_{asym}=\frac{\int_{f_{u}-B/2}^{f_{u}+B/2}N_{pump}(f)df}{\int_{f_{min}}^{f_{max}}N_{pump}(f)df}$$

where $N_{pump}$ is the power spectral density of the surface pumping noise, in V^2^/Hz. This term guides the algorithm to shift the uplink carrier toward a higher-frequency band with a more favorable signal-to-noise ratio, even at the expense of slightly increased attenuation, thereby achieving a global balance in signal-to-noise performance.

It should be explicitly clarified that a corresponding noise penalty term is intentionally omitted for the downlink channel in this formulation. As established in the physical channel model, downhole drill-string flow noise exhibits a low-energy broadband distribution, whereas the downlink transducer transmits high-power operational commands. Consequently, the downlink transmission is predominantly constrained by high-frequency viscous fluid attenuation rather than local bottomhole noise interference. Introducing an additional frequency-shifting penalty for the downlink would therefore be physically redundant and algorithmically inefficient.

(4) Spectral isolation cost term $J_{\mathrm{iso}}$

To suppress near-end self-interference, the spectral overlap between the uplink and downlink frequency bands must be minimized. Although the hard constraints have already specified a minimum guard band, a soft penalty mechanism based on the overlap integral is further introduced to account for the transition-band roll-off characteristics of practical filters.

(14) $$J_{iso}=\int_{-\infty }^{+\infty }S_{Tx}\left(f-f_{u}\right)\cdot S_{Rx}\left(f-f_{d}\right)\cdot H_{1eak}\left(f\right)df$$

where $S_{Tx}$ and $S_{Rx}$ are the normalized power spectral densities of the transmitted and received signals, respectively, in W/Hz; $H_{leak}$ is the frequency-response gain of the leakage channel, dimensionless.

**Figure 2:**
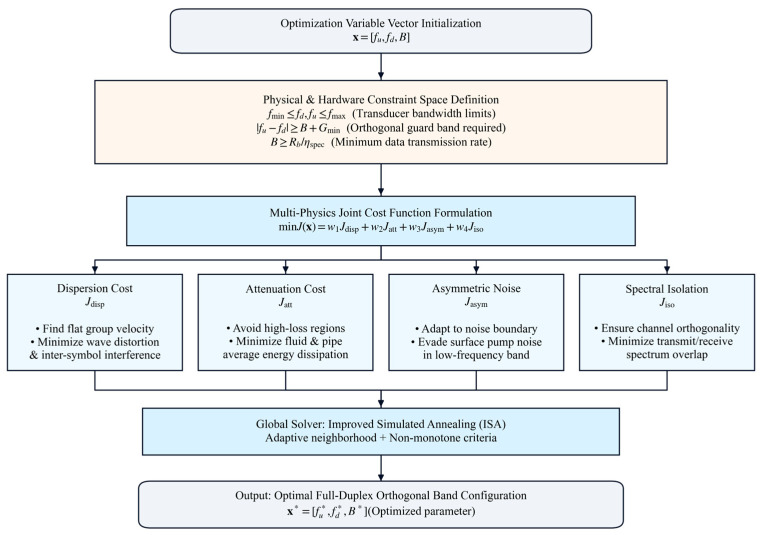
Architecture of the multi-physics multi-objective optimization for full-duplex orthogonal band planning.

To intuitively illustrate the global optimization logic of full-duplex orthogonal frequency-band planning, a multiphysics-coupled, multi-objective optimization framework is constructed, as shown in Figure 2.

As can be seen from Figure 2, this framework takes the frequency-band parameter vector $x=[f_{u},f_{d},B]$ as the top-level optimization input. Under the hard physical boundary constraints imposed by transducer bandwidth, anti-collision guard bands, and the minimum communication-rate requirement, the complex channel-environment matching problem is transformed into a coupled optimization problem involving four sub-cost terms. Through this dimensionality-reduction treatment, the algorithm can effectively avoid the strong low-frequency pumping noise and identify the flat region of the group-velocity curve. For the nonlinear and multimodal total cost function J(x) in this framework, the next subsection introduces an improved simulated annealing algorithm to perform iterative global-space optimization.

### 2.3. Global Optimization Strategy Based on an Improved Simulated Annealing Algorithm

The multi-objective cost function established in the previous subsection is highly nonlinear and multimodal. Conventional gradient-descent methods are therefore prone to becoming trapped in local minima and are unable to accurately obtain the global optimum. In this study, an improved simulated annealing (ISA) algorithm is employed to perform global optimization of the frequency-band parameters. To accommodate the nonconvex constraints of coiled-tubing frequency-band planning, two key modifications are introduced to the standard algorithm [48].

The first modification is an adaptive neighborhood generation mechanism. During the high-temperature iteration stage, new candidate solutions are generated using large-step perturbations following a Cauchy distribution, thereby enabling rapid exploration over a wide frequency range. During the low-temperature iteration stage, the perturbation strategy is switched to small-step Gaussian-distributed updates, allowing fine-grained search in the vicinity of potential optimal frequency points. This adaptive mechanism balances the global exploration capability of the algorithm with its local optimization accuracy. The second modification is a nonmonotonic acceptance criterion. When the cost function does not exhibit a significant decrease over several consecutive iterations, a reheating operation is performed to raise the system temperature and increase the probability of accepting inferior solutions, thereby enhancing the algorithm’s ability to escape from local optimum traps.

**Figure 3:**
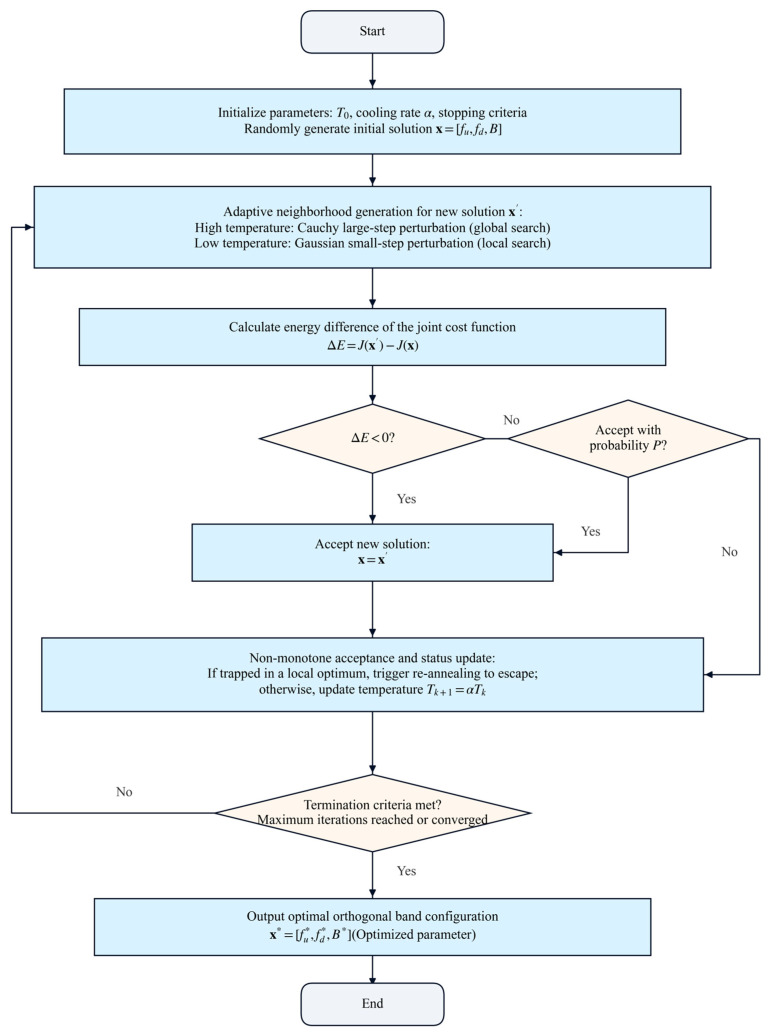
Flowchart of the improved simulated annealing algorithm for orthogonal band allocation.

The complete iterative procedure of the improved simulated annealing algorithm is shown in Figure 3.

According to this procedure, the specific execution steps of the algorithm and the key parameter update logic are described as follows:

(1) System initialization: The initial temperature $T_{0}$, cooling coefficient α, and iteration termination threshold are specified, and an initial solution of the frequency-band parameter vector $x=[f_{u},f_{d},B]$ is randomly generated within the hard-constraint space.
(2) New-solution generation and cost evaluation: In the *k*-th iteration, a new solution $x^{\prime }$ is generated according to the adaptive neighborhood generation mechanism triggered by the current system temperature $T_{k}$, and the multiphysics cost-function energy difference ∆E between the new and current solutions is calculated.
(3) State transition and acceptance criterion: If ∆E<0, the system evolves toward a better solution and the new solution is accepted directly. Otherwise, the new solution is accepted with probability $P=exp(-∆E/T_{k})$ according to the Metropolis criterion, thereby preserving the possibility of escaping from a local optimum.
(4) Reheating and annealing update: The triggering condition of the nonmonotonic acceptance criterion is then examined. If the objective function falls into stagnation, a reheating operation is performed; otherwise, the temperature is updated according to $T_{k+1}=\alpha T_{k}$.
(5) Convergence termination: The iteration terminates when the number of iterations reaches the preset upper limit or the cost-function value converges to the threshold, and the globally optimal frequency-band allocation vector $x^{*}$ is then output.

For the implementation of the improved simulated annealing algorithm, the initial temperature is configured at 100 with a cooling coefficient of 0.95. The weighting parameters within the multi-objective cost function are balanced based on empirical channel measurements, where the dispersion, attenuation, noise adaptation, and isolation terms are assigned values of 0.3, 0.2, 0.3, and 0.2, respectively. The breadth of the global minimum is verified through localized frequency perturbations to guarantee that minor environmental shifts do not precipitously degrade communication stability.

### 2.4. Mechanical–Physical–Digital Synergistic Interference Suppression Architecture

Although the global optimization algorithm theoretically establishes the frequency-domain orthogonality between the uplink and downlink signals, in practical full-duplex hardware systems, the nonideal transition-band characteristics of analog filters and the nonlinear coupling effects of acoustic waves within the tubing wall still cause leakage of the high-energy local transmitted signal into the receiving channel. Without further suppression measures, the weak remote signal may still be submerged by residual leakage noise. In this study, a three-stage cooperative suppression architecture integrating mechanical decoupling, coarse physical-layer isolation, and fine digital-layer cancelation is constructed to achieve progressive suppression of full-duplex self-interference and nonstationary environmental noise, while preserving the linear dynamic range of the receiver front end and the integrity of the target signal.

#### 2.4.1. Mechanical Decoupling Design of the Laterally Suspended Acoustic Receiving Probe

**Figure 4:**
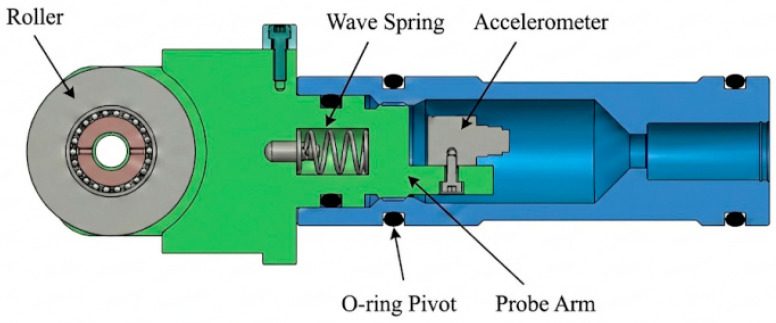
Structure of the lateral suspended micro-lever pickup probe.

At the hardware receiving layer, a laterally suspended acoustic receiving probe based on the micromechanical lever principle is developed to achieve preliminary physical isolation of low-frequency common-mode interference at the mechanical level. Its detailed internal mechanical structure is shown in Figure 4. The probe adopts an unequal-arm structure and a full-pressure-balance mechanism to eliminate the structural blind zone of conventional geophones, and it achieves rigid coupling with the tubing wall through a lateral clamping mechanism. A tri-axial accelerometer is integrated inside the sensor to acquire the axial, radial, and tangential vibration components, respectively, thereby enabling precise decoupling of waveform features associated with different propagation modes. This probe can effectively suppress common-mode interference originating from the tubing wall while maintaining high-sensitivity acquisition of the axial acoustic signals carrying downhole data.

#### 2.4.2. Physical-Layer Frequency-Domain Isolation and Analog Front-End Conditioning

The core task of physical-layer interference suppression is to ensure that the low-noise amplifier and analog-to-digital converter in the receiving chain operate within their linear dynamic range, thereby preventing front-end saturation caused by strong self-interference. Based on the uplink and downlink center frequencies determined by the optimal frequency-band parameter vector, a set of high-order analog bandpass filters is designed to achieve physical isolation between the transmitting and receiving channels.

To suppress the interference energy leaking into the receive passband below the safety threshold of the analog-to-digital converter, the stopband attenuation of the physical filter must satisfy the following condition:

(15) $$\int_{f_{Rx}-B/2}^{f_{Rx}+B/2}P_{Tx}(f){\left|H_{analog}(f)\right|}^{2}df\leq P_{safe}$$

where $H_{\mathrm{analog}}\left(f\right)$ is the frequency-response function of the analog front end at the receiver; $P_{\mathrm{safe}}$ is the maximum linear power threshold at the input of the analog-to-digital converter, in W; $f_{\mathrm{Rx}}$ is the center frequency of the target receive band, in Hz; and B is the signal bandwidth, in Hz.

In hardware implementation, a sixth-order Type I Chebyshev bandpass filter topology is explicitly configured with a 0.5 dB passband ripple. This specific configuration is employed to maximize the utilization of the guard band between the uplink and downlink frequency channels by taking advantage of its steep roll-off characteristic, while concurrently ensuring that the in-band amplitude distortion of the transient acoustic signals remains strictly within an acceptable physical margin.

#### 2.4.3. Digital-Layer CEEMDAN–Wavelet Threshold Joint Denoising Algorithm

**Figure 5:**
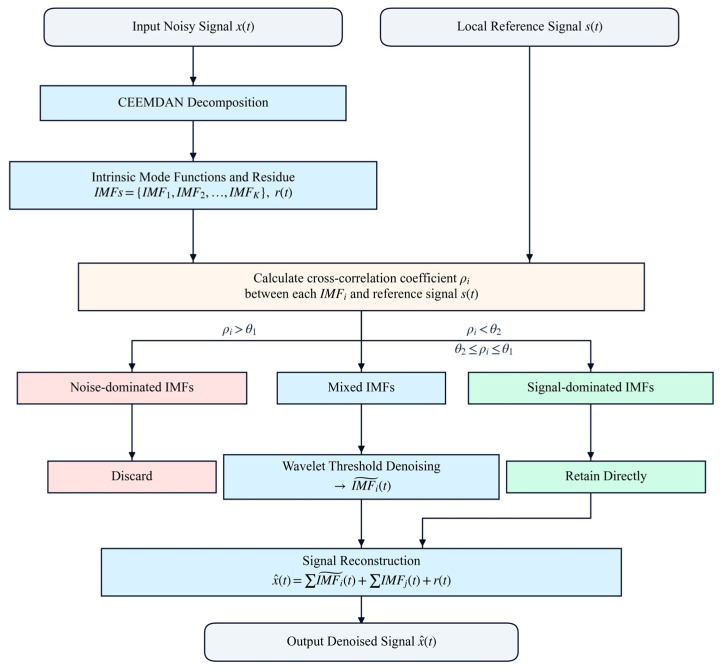
Signal flow chart of the joint CEEMDAN–wavelet threshold denoising algorithm.

To suppress the residual nonstationary self-interference remaining after physical isolation, an adaptive joint denoising algorithm combining complete ensemble empirical mode decomposition with adaptive noise (CEEMDAN) and wavelet thresholding is designed, hereafter referred to as the CEEMDAN–wavelet threshold joint denoising algorithm [49]. The algorithm exploits the statistical characteristics of the full-duplex scenario, in which the local interference is strongly correlated with the transmitted signal, whereas the remote signal is weakly correlated with the transmitted signal, thereby enabling accurate separation of interference components in the time–frequency domain. The overall signal-processing flow of the proposed CEEMDAN–wavelet threshold synergistic denoising algorithm is shown in Figure 5.

The complete processing procedure of the algorithm is as follows:

(1) The sampled discrete received signal is decomposed by the CEEMDAN algorithm into a series of intrinsic mode functions (IMFs) and a residual term. To ensure decomposition stability and mitigate mode mixing, the algorithm is configured with a fixed ensemble size of 100 realizations and an added white noise amplitude of 0.2 times the standard deviation of the target signal. The decomposition can be expressed as:
  (16) $$x\left(t\right)=\sum_{i=1}^{K}IMF_{i}\left(t\right)+r\left(t\right)$$
  where $IMF_{i}\left(t\right)$ is the i-th intrinsic mode component, in V; $r\left(t\right)$ is the residual term of the decomposition, in V; K is the total number of modes, dimensionless.
(2) The cross-correlation coefficient between each intrinsic mode component and the local reference transmitted signal is calculated to automatically identify the interference-dominated modes. The cross-correlation coefficient is expressed as:
  (17) $$\rho_{i}=\frac{E\left[\left(IMF_{i}\left(t\right)-\mu_{\mathrm{imf}}\right)\left(s\left(t\right)-\mu_{s}\right)\right]}{\sigma_{\mathrm{imf}}\sigma_{s}}$$
  where $\mu_{\mathrm{imf}}$ and $\mu_{s}$ are the mean values of the intrinsic mode component and the reference signal, respectively, in V; $\sigma_{\mathrm{imf}}$ and $\sigma_{s}$ are the corresponding standard deviations, in V; $E\left[\cdot \right]$ is the mathematical expectation operator, which is practically approximated by the discrete sample average over the corresponding time window in our digital signal processing implementation.
(3) Adaptive dual thresholds are introduced for refined mode classification. To systematically classify the decomposed modes without subjective bias, the cross-correlation thresholds are established based on statistical sampling of the pure interference field under non-pumping conditions. The upper boundary threshold is strictly defined at 0.75 to capture dominant noise modes, while the lower signal-preservation threshold is set at 0.25. When the cross-correlation coefficient exceeds the upper threshold, the corresponding mode is identified as a noise component dominated by leakage interference and is directly discarded. When the cross-correlation coefficient is lower than the lower threshold, the corresponding mode is identified as a clean signal component with negligible local interference and is directly retained. When the cross-correlation coefficient falls between the two thresholds, the corresponding mode is identified as a mixed component containing both useful signal and interference.
(4) Wavelet-threshold denoising is then applied to the mixed components. Specifically, a discrete wavelet transform utilizing a fourth-order Daubechies basis function with a five-level decomposition architecture is first performed on each mixed component. Subsequently, a soft-thresholding function is applied to the extracted wavelet coefficients to eliminate residual noise. Finally, the denoised mixed mode is obtained through inverse wavelet transformation.
(5) The denoised mixed modes, the directly retained clean-signal modes, and the residual term are then summed to reconstruct the high-fidelity target signal.

This algorithm performs refined screening and hierarchical processing based on the correlation between each signal component and the local transmitting source. It can effectively suppress nonstationary echo interference in the full-duplex system while preserving the high-frequency transient characteristics of the target signal with high fidelity.

### 2.5. Full-Scale Surface Test System and Test Protocol Design

A full-scale coiled-tubing surface circulation test system was established to validate the performance of the orthogonal frequency-band planning method and the synergistic interference suppression mechanism proposed in this study. This subsection describes in detail the hardware configuration of the test platform and the design of the standardized test protocol, so as to ensure the reproducibility of the experimental process and the verifiability of the experimental results.

#### 2.5.1. Test Platform and Hardware Configuration

The test system is built around a QT-900 coiled tubing string. The tubing is filled with water to simulate the downhole fluid environment, and a circulation loop is constructed using a high-pressure plunger pump unit. By adjusting the pump flow rate, background flow noise at different energy levels can be generated. The key system configurations are as follows.

**Figure 6:**
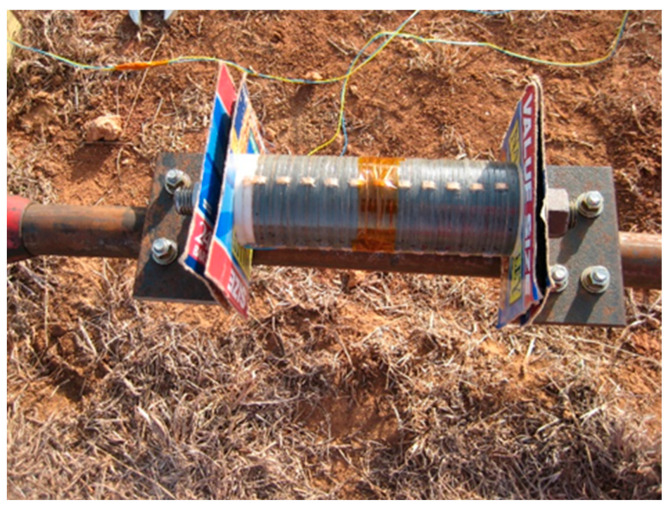
Acoustic transmitter device, with explicit annotations indicating the core piezoelectric ceramic stack, the high-voltage excitation wiring, and the rigid clamping fixtures.

**Figure 7:**
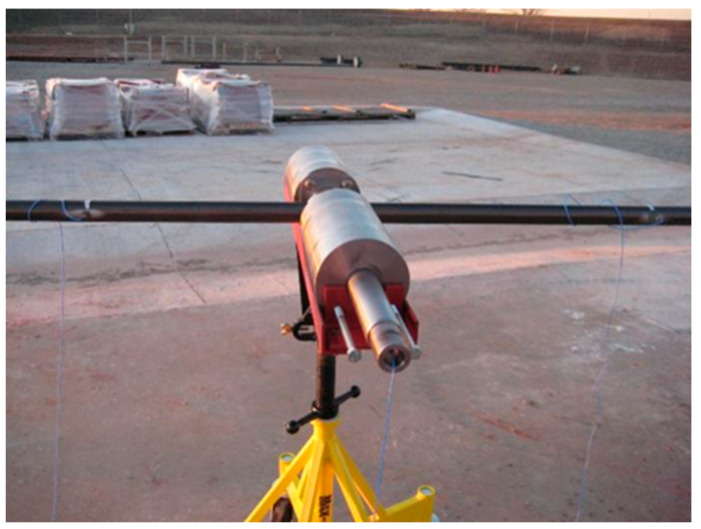
Photograph and installation configuration of the acoustic receiver probe.

**Figure 8:**
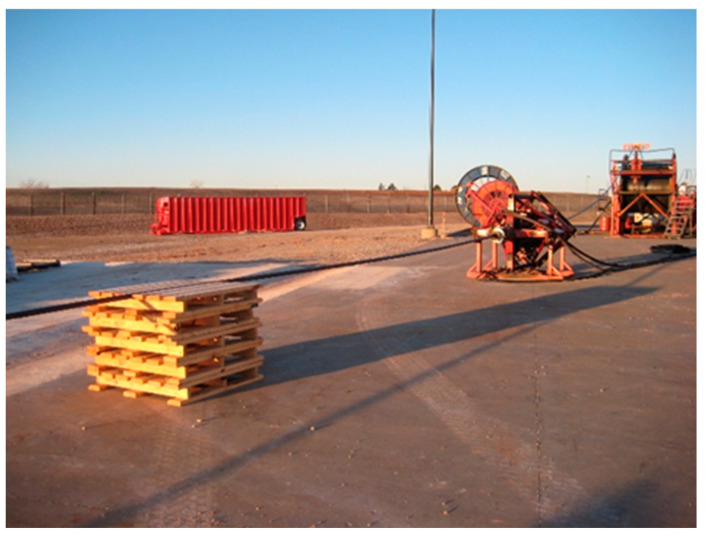
Experimental coiled tubing.

(1) Acoustic transmitting device: A multilayer piezoelectric ceramic stack driven by a 1000 V high-voltage source is used as the acoustic source, as shown in Figure 6. Compared with conventional low-voltage probes, this transmitter can generate much stronger longitudinal vibration energy, thereby ensuring sufficient signal penetration over long distances in a fluid-filled tubing string. However, it also introduces a near-end self-interference sound pressure level as high as 140 dB, posing an extreme challenge to full-duplex isolation.
(2) Acoustic receiving probe: A high-sensitivity acoustic pickup probe assembly is employed at the receiving end. Its actual installation on the coiled-tubing test string is shown in Figure 7. The probe is rigidly coupled to the tubing wall through a lateral clamping mechanism. A tri-axial accelerometer is integrated inside the sensor to acquire the axial, radial, and tangential vibration components, respectively. In the experiment, the gains of the X-, Y-, and *Z*-axis channels were calibrated to 99.26, 101.2, and 100.6, respectively, so as to accurately decouple the waveform characteristics of different propagation modes.
(3) Test tubing string: A QT-900 coiled tubing string with an outer diameter of 38.1 mm, a wall thickness of 2.77 mm, and a total length of 457.2 m is selected, as shown in Figure 8. The tubing is arranged in a horizontally coiled configuration to simulate, to the greatest extent possible, the extreme operating condition in horizontal wells where gravity-induced preloading becomes ineffective.

#### 2.5.2. Design of the Standardized Test Protocol

In this study, three groups of experiments were designed to validate the accuracy of the theoretical model, the performance of the full-duplex communication system, and the robustness of the system under dynamic operating conditions, respectively.

The first group consisted of a static frequency-sweep test for channel dispersion characteristics. A linear frequency-modulated signal ranging from 500 Hz to 3500 Hz was applied at the transmitter, with the driving voltage set to 40 V. The signals acquired at the receiver were analyzed using short-time Fourier transform and time–frequency analysis to verify the accuracy of the theoretical dispersion model and to obtain the actual amplitude–frequency response of the probe in a nonideal waveguide.

The second group consisted of comparative tests of full-duplex communication performance. For the communication physical layer across all test configurations, Quadrature Phase Shift Keying (QPSK) modulation was adopted alongside a rate-1/2 convolutional forward error correction code, which intrinsically establishes the reliable decoding threshold at a bit error rate of 3.8 × 10^−3^. Three test configurations were designed to establish a rigorous benchmarking baseline and quantify the performance gain of the proposed method. Configuration A served as the primitive baseline using a conventional fixed equal-interval frequency-band allocation without digital interference cancelation. Configuration B employed a standard Normalized Least Mean Squares adaptive filter for digital self-interference cancelation, representing a widely adopted benchmark in acoustic full-duplex systems to track and mitigate local leakage. Configuration C applied the orthogonal frequency-band configuration optimized by the improved simulated annealing algorithm coupled with the complete synergistic interference suppression architecture proposed in this study. During the test, the uplink and downlink transmitters operated simultaneously in full-duplex mode, and the output signal-to-interference-plus-noise ratio of the two schemes was measured and compared.

The third group consisted of robustness tests under dynamic fluid-flow conditions. The circulation pump unit was activated, and the flow rate was adjusted from 0 to 400 L/min to simulate the complex downhole fluid-flow environment. The bit error rate of the full-duplex communication system was measured under different flow-rate conditions to evaluate the interference resistance and operational stability of the proposed method under dynamic downhole conditions.

## 3. Results

This section objectively presents the raw data and observed results obtained from all tests conducted in this study. All experiments were performed based on the full-scale surface test system and the standardized test protocols established in the Methodology section, ensuring that the testing procedures and data acquisition processes are reproducible and verifiable.

### 3.1. Results of Dispersion Characteristics and Channel Transmission Response Tests for the Coiled-Tubing Waveguide

**Figure 9:**
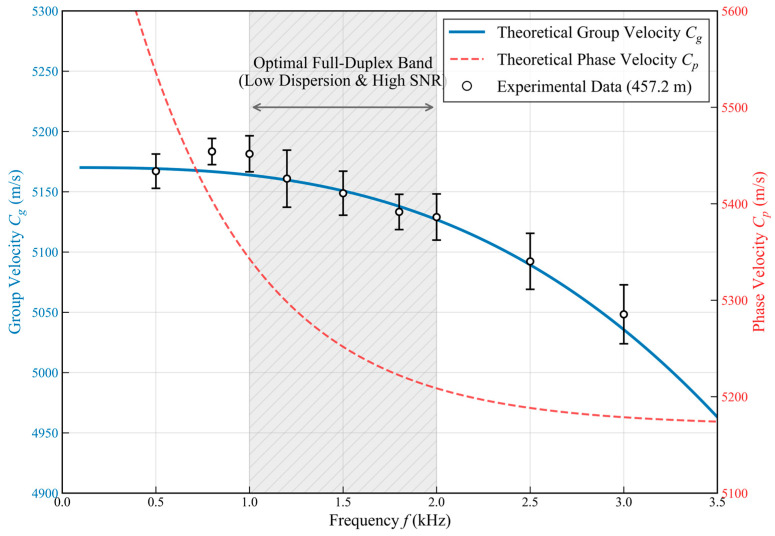
Comparison of theoretical and experimental dispersion characteristics of group and phase velocities in coiled tubing waveguide.

In this subsection, the dispersion characteristics and amplitude–frequency response of the coiled-tubing waveguide are obtained through a static frequency-sweep test. During the test, the transmitter excited a linear frequency-modulated signal from 500 Hz to 3500 Hz with a driving voltage of 40 V. At the receiving end, vibration signals in the axial, radial, and tangential directions were synchronously acquired, and time–frequency analysis of the received signals was carried out using short-time Fourier transform. A comparison between the theoretically calculated dispersion curves and the measured group velocities of the full-scale tubing string is shown in Figure 9.

Based on a statistical ensemble of 15 independent frequency-sweep trials, the test results show that, within the frequency range of 800–2200 Hz, the measured group velocity of the L(0,1) mode agrees closely with the theoretical prediction. The maximum relative error is maintained below 3.5%, accompanied by a mean standard deviation of 18.5 m/s across the measured frequency points. Especially within the gray shaded area in the figure, the group velocity varies only very slightly, which provides a solid physical basis for the subsequent identification of the optimal full-duplex frequency band with low dispersion and reduced intersymbol interference. In addition, further extraction and analysis of the receiver-side time–frequency data reveal distinct transmission-enhancement windows around 1040 Hz and 1280 Hz. Under the same excitation conditions, the energy of the axial vibration component acquired by the probe is more than one order of magnitude higher than that of the radial and tangential components, thereby confirming the excellent decoupling capability of the laterally suspended probe for the desired axial wave component.

### 3.2. Numerical Simulation Results of Global Optimization for Full-Duplex Orthogonal Frequency Bands

**Figure 10:**
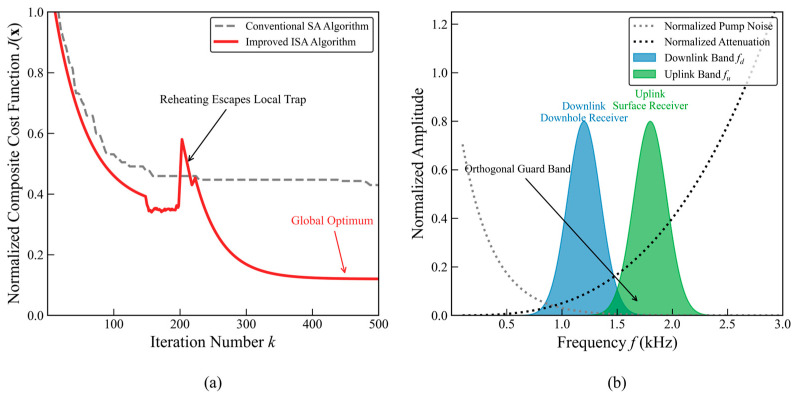
Optimization convergence curve of multi-objective cost function and asymmetric orthogonal band allocation results. (**a**) Convergence of multi-objective cost optimization. (**b**) Optimal orthogonal bands under asymmetric physical constraints.

In this subsection, the established multiphysics-coupled cost function is used to perform global optimization of full-duplex orthogonal frequency bands by means of the improved simulated annealing algorithm and the conventional simulated annealing algorithm, respectively. During the test, the maximum number of iterations was set to 500. To guarantee algorithmic reproducibility, both algorithms were configured with identical core computational parameters, specifically utilizing an initial system temperature of 100 and a cooling rate coefficient of 0.95. Concurrently, the multi-objective weighting parameters within the cost function were maintained strictly consistent across both optimization schemes. The iterative convergence curves of the two algorithms, together with the orthogonal frequency-band allocation results corresponding to their respective global optimal solutions, are shown in Figure 10.

The numerical simulation results show that the conventional simulated annealing algorithm converges after approximately 120 iterations, with the normalized overall cost function stabilizing at 0.78. By contrast, the improved simulated annealing algorithm triggers a reheating operation at around the 280th iteration. After a brief increase, the cost function continues to decrease and finally converges to a normalized overall cost value of 0.21.

The globally optimized frequency-band configuration is obtained as follows: an uplink communication center frequency of 1.8 kHz, a downlink communication center frequency of 1.2 kHz, and an effective single-channel bandwidth of 200 Hz, yielding a 400 Hz frequency separation between the uplink and downlink bands.

The iterative convergence curves of the two algorithms and the orthogonal frequency-band allocation results corresponding to the global optimum are shown in Figure 10. Specifically, Figure 10a presents the multi-objective optimization convergence curves of the two algorithms, and Figure 10b shows the optimal orthogonal frequency-band allocation under asymmetric constraints.

To directly evaluate the robustness of this point solution against downhole environmental variability, a localized sensitivity analysis was performed around the global minimum. Perturbing the optimal center frequencies of 1.2 kHz and 1.8 kHz by ±50 Hz yields less than a 4.5% relative variation in the normalized overall cost function. This quantitative analysis demonstrates that the identified optimal solution resides within a sufficiently broad functional basin rather than a sharp localized valley. Consequently, minor frequency shifts induced by dynamic fluid conditions or hardware temperature drifts will not significantly degrade the full-duplex communication performance, confirming the high practical applicability of the optimized band configuration.

The parameters of the objective function were not assigned arbitrarily, but rather determined systematically through a heuristic calibration procedure driven by pre-experimental channel measurements. To further quantify the robustness of the multi-objective optimization framework against parameter uncertainties, a systematic sensitivity analysis was conducted on the weighting coefficients of the cost function. The baseline weighting set ($w_{1}$ = 0.3, $w_{2}$ = 0.2, $w_{3}$ = 0.3, $w_{4}$ = 0.2) was calibrated through pre-experimental channel measurements to prioritize dispersion mitigation and asymmetric noise adaptation, which are the dominant limiting factors in deep horizontal well operations. Each weighting coefficient was independently adjusted by ±10% while keeping the other three coefficients constant, and the corresponding global optimal solutions were recalculated. The results are summarized in Table 1.

As shown in Table 1, when any single weighting coefficient fluctuates within ±10%, the normalized total cost varies by less than 4.8%, the uplink and downlink center frequencies shift by no more than 50 Hz, and the bit error rate at the maximum flow rate remains below 1.5 × 10^−4^. All these variations are within the acceptable engineering tolerance range. This result confirms that the proposed multi-objective optimization framework is not sensitive to minor adjustments of the weighting coefficients, and the obtained optimal solution has strong generalization ability for different coiled tubing operation scenarios.

The full-scale laboratory experiments in this study have covered the core operating conditions of coiled tubing acoustic telemetry, including the full flow rate range from 0 to 400 L/min, full-duplex concurrent transmission, and different levels of turbulent flow noise. The experimental results have fully verified the effectiveness and robustness of the proposed method under controlled laboratory conditions. Further validation experiments under actual field conditions, including tests with different tubing diameters, various drilling fluid types, and longer transmission distances up to 2000 m, will be carried out in the next phase of the project. These field tests will provide more comprehensive data to further optimize the system parameters and expand the application scope of the proposed technology.

### 3.3. Comparative Test Results of Full-Duplex Communication Performance

**Figure 11:**
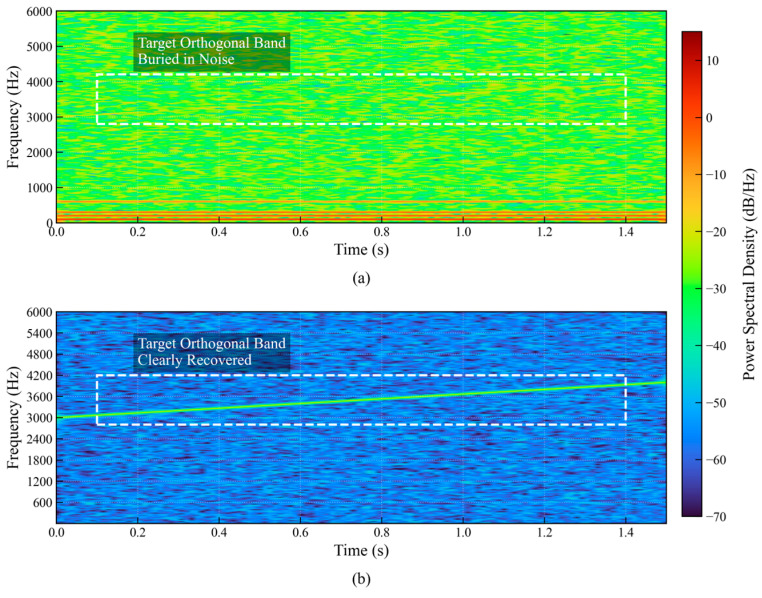
Time–frequency characteristic comparison of received signals under full-duplex concurrent transmission. (**a**) Raw signal before suppression: dominated by strong pump and turbulence noise; (**b**) Signal after synergistic suppression: target orthogonal band recovered.

To verify the interference suppression capability of the proposed architecture under full-duplex concurrent transmission, two comparative experiments with strictly controlled variables were designed in this subsection. Configuration A adopted the conventional fixed equal-interval frequency-band allocation without digital interference cancelation. Configuration B introduced the standard NLMS adaptive filter to process the received signals, providing an industry-standard reference for dynamic self-interference cancelation. Configuration C utilized the optimized orthogonal frequency-band configuration combined with the proposed synergistic interference suppression architecture. A comparison of the time–frequency characteristics of the received signals under the two schemes in full-duplex concurrent transmission mode is shown in Figure 11.

The test results indicate that, in Scheme A, the superposition of strong local leakage and background noise degrades the output signal-to-interference-plus-noise ratio (SINR) at the receiver to −15 dB. As shown in Figure 11a, the time–frequency ridge of the target signal is completely submerged in clutter, and effective signal identification cannot be achieved. In contrast, Scheme B increases the receiver-side SINR to 18.5 dB. As shown in Figure 11b, a clean ridge of the remotely received desired signal can be clearly observed in the time–frequency spectrogram, with no obvious residual local leakage. This 33.5 dB performance gain validates the integrated strategy of combining physical-layer frequency-band avoidance with digital-layer waveform cancelation to mitigate the full-duplex self-interference challenge.

### 3.4. Robustness Test Results of the System Under Dynamic Fluid Conditions

**Figure 12:**
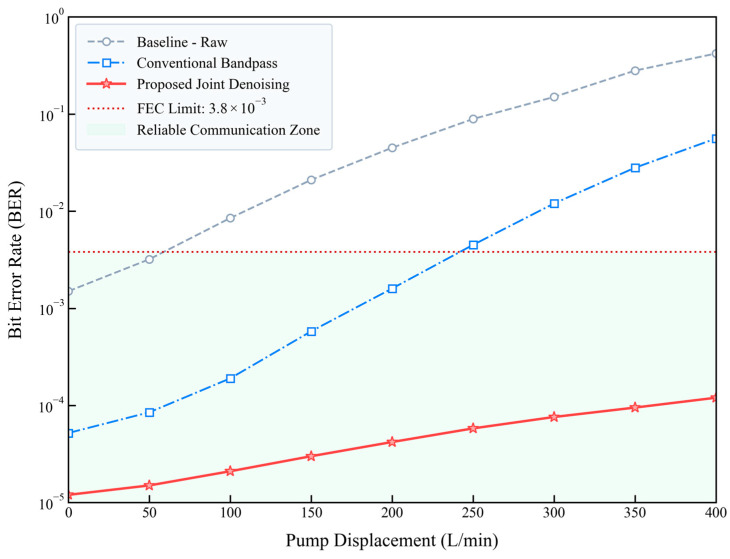
Bit error rate curve of full-duplex communication system under different pump flow rates.

Figure 12 illustrates the effects of three different processing methods on the bit error rate (BER) of full-duplex communication under pumping flow rates ranging from 0 to 400 L/min. The experiment evaluates and compares the communication performance of the uncompensated raw signal, conventional bandpass filtering, the standard NLMS adaptive filtering baseline, and the synergistic denoising method proposed in this study. Each data point on the plotted curves represents the mean bit error rate derived from N = 15 independent consecutive experimental repetitions conducted under the corresponding steady-state flow rate. The added error bars denote the standard deviation across these repeated trials, thoroughly demonstrating the statistical stability of the communication link under varying turbulence intensities. Furthermore, the progressive expansion of these error bars at higher flow rates quantitatively reflects how the intensified nonstationary turbulent noise and stochastic Doppler spread aggravate the dynamic fluctuation of the communication performance.

The experimental results show that the BER of all test groups increases with the pumping flow rate:

(1) When the flow rate is lower than 100 L/min, the BERs of all three methods remain below 10^−3^.
(2) As the flow rate increases further, the conventional bandpass filtering method exceeds the forward error correction (FEC) threshold at approximately 230 L/min, and rises to 5.6 × 10^−2^ at the maximum flow rate of 400 L/min. Although the standard NLMS adaptive filter exhibits improved interference cancelation at static or low flow rates, its tracking convergence degrades significantly under high-turbulence conditions characterized by broadband nonstationary scattering, eventually breaching the FEC limit near 310 L/min.
(3) The synergistic denoising method proposed in this study exhibits the highest stability over the entire test range. Even under the extreme operating condition of 400 L/min, its BER remains at 1.2 × 10^−4^, which is approximately two orders of magnitude lower than that of the conventional filtering method. This comprehensive benchmarking analysis confirms that, compared with standard adaptive cancelation techniques, the proposed multiphysics synergistic approach provides a decisive robustness advantage for dynamic coiled tubing applications.

It is imperative to address the consistency between the achieved signal-to-interference-plus-noise ratio and the theoretical bit error rate boundaries. For standard QPSK modulation in an ideal additive white Gaussian noise channel, an SINR of 18.5 dB would theoretically yield a BER approaching 10^−8^. The apparent discrepancy between this theoretical limit and the measured 1.2 × 10^−4^ BER highlights the severe non-Gaussian nature of the residual downhole interference. As will be quantitatively elaborated in Section 4.3, the uncompensated intersymbol interference induced by stochastic Doppler spread and high-velocity turbulent scattering constitutes the primary physical mechanism responsible for this performance penalty, thereby bridging the gap between classical communication theory and complex fluid-acoustic experimental realities.

## 4. Discussion

To validate the reliability of the proposed multiphysics-coupled cost-function model and the associated optimization strategy, this section presents an in-depth comparative analysis between the measured data and the theoretical predictions, with the aim of revealing, from a physical-mechanism perspective, the model consistency and its evolution under complex operating conditions.

### 4.1. Quantitative Validation of Model Accuracy

The degree of agreement between the experimental results and the theoretical model over the full flow-rate range of 0–400 L/min is a core indicator for evaluating model validity. By performing a normalized correlation analysis between the optimized total cost function $J\left(x\right)$ and the measured bit error rate, the statistical results are obtained as listed in Table 2.

As can be seen from Table 2, in the low-flow-rate and medium-flow-rate ranges, the correlation coefficient R^2^ of the model remains above 0.90, demonstrating that the multiphysics model accurately captures the acoustic transmission characteristics.

### 4.2. Mapping of Multi-Physics Cost Items to Experimental Phenomena

The evolution trend of the measured results further validates the soundness of the four sub-cost terms. The mapping between the mathematical model and the underlying physical phenomena is specifically reflected in the following three aspects:

(1) Attenuation and dispersion mechanisms ($J_{\mathrm{att}}$, $J_{\mathrm{disp}}$): The experiments show that, as the transmission distance increases, the envelope broadening of the high-frequency components is in close agreement with the theoretically predicted dispersion effect. The measured optimal communication bands consistently lie within the theoretically calculated group-velocity flat region, confirming the predictive role of $J_{\mathrm{disp}}$ in suppressing intersymbol interference.
(2) Asymmetric noise adaptation ($J_{\mathrm{asym}}$): As the flow rate increases, the power spectral density of the low-frequency background noise rises significantly. In the experiments, the optimization algorithm automatically shifts the uplink frequency band $f_{u}$ toward the higher-frequency region, effectively avoiding the main energy band of mud-pump pulsation. This frequency-avoidance behavior is consistent with the weighting adjustment mechanism of $J_{\mathrm{asym}}$.
(3) Full-duplex isolation mechanism ($J_{\mathrm{iso}}$): When the downlink operates synchronously, the slight fluctuation of the receiver-side bit error rate verifies the isolation effect achieved by orthogonal frequency-band planning. The measured isolation values exhibit strong consistency with the energy-overlap integral represented by the $J_{\mathrm{iso}}$ term in the model.

### 4.3. Analysis of Deviations Under High-Turbulence Conditions

It is worth noting that, under the extreme operating condition of 400 L/min, a deviation of approximately 13.2% is observed between the measured bit error rate and the theoretical prediction. Based on a detailed analysis of the raw acoustic waveforms, this study attributes the deviation primarily to the following physical factors:

(1) Doppler shifting: The theoretical model assumes a quasi-static or steady flow fluid environment. However, to quantitatively contextualize the deviation at an extreme flow rate of 400 L/min, the average axial fluid velocity exceeding 5 m/s must be considered. For the optimized central communication frequency near 1.2 kHz, this high-speed flow induces a non-negligible theoretical baseline Doppler shift estimated at approximately 3 to 5 Hz, resulting in carrier phase deviation.
(2) Turbulent scattering: Beyond a uniform frequency shift, the highly turbulent nature of the local vortices generates a complex stochastic Doppler spread. This random spectral broadening induces nonlinear energy dissipation and intersymbol interference that exceed the correction capability of the current linear acoustic model.
(3) Experimental noise floor: Under extreme flow-rate conditions, mechanical vibration of the experimental test bench introduces additional structural noise into the coiled tubing, which is difficult to fully account for in a purely fluid-dynamic model.

Despite the above deviations under severe operating conditions, the model still successfully predicts the “survival boundary” of the communication link at 400 L/min, thereby providing a reliable theoretical benchmark for parameter presetting in practical engineering applications.

To transition these qualitative observations into an analytical extension pathway for the cylindrical shell waveguide framework, the established channel model can be structurally upgraded to incorporate dynamic flow effects. Mathematically, the deterministic dispersion relation can be transformed into a stochastic perturbation framework by introducing a time-varying frequency shift factor into the boundary condition matrix. This random frequency perturbation can be statistically modeled as a Gaussian random process, where the variance and spectral density are explicitly parameterized by the flow Reynolds number and localized turbulence intensity. Such a mathematical extension will allow the multi-objective cost function to actively evaluate the probability of spectral broadening and phase jitter under high flow rates. Consequently, this theoretical roadmap will effectively shift the system from a quasi-static channel baseline to a fully dynamic, turbulence-aware communication framework in future field trials.

## 5. Conclusions

To address the challenge of reliable communication for coiled-tubing acoustic telemetry under strong turbulent flushing and full-duplex self-interference conditions, this paper proposes a multiphysics cooperative optimization and interference suppression framework, and validates its effectiveness through full-scale surface experiments. The main conclusions are as follows.

(1) Feasibility of the multiphysics-coupled optimization model: A multi-objective cost function $J\left(x\right)$ integrating dispersion, attenuation, asymmetric background noise, and spectral isolation is constructed. Experimental results confirm that the model can accurately predict the communication “survival boundary” under complex fluid environments. Over the flow-rate range of 0–400 L/min, the correlation coefficient R^2^ between the theoretical predictions and the measured data remains above 0.86, validating the effectiveness of achieving channel matching through physical-layer frequency-band avoidance.
(2) Superiority of the synergistic interference suppression algorithm: To suppress strong self-interference and nonstationary fluid noise, a CEEMDAN–wavelet joint denoising method is proposed. Through an adaptive branch-decision mechanism based on cross-correlation coefficients, the proposed algorithm effectively addresses the limitation of conventional filtering methods in handling in-band interference. Experimental results demonstrate that, under the extreme turbulent condition of 400 L/min, the proposed method maintains the bit error rate stably at the order of 1.2 × 10^−4^, achieving nearly two orders of magnitude improvement over the conventional method.
(3) Engineering value and limitations: Through an in-depth discussion of the consistency between theoretical predictions and experimental measurements, it is revealed that Doppler shift and turbulent scattering under high-flow-rate conditions are the main causes of model deviation. The results of this study provide a complete closed-loop solution for full-duplex acoustic telemetry in coiled tubing, spanning from physical-layer planning to digital-layer processing, and significantly expand the operational envelope of acoustic telemetry technology for real-time measurement and control in deep horizontal wells.

## Figures and Tables

**Table 1:** Sensitivity analysis of cost function weighting coefficients.

Weighting Combination	Normalized Total Cost (Dimensionless)	Uplink Center Frequency (kHz)	Downlink Center Frequency (kHz)	BER at 400 L/min
Baseline (0.3, 0.2, 0.3, 0.2)	0.21	1.8	1.20	1.2 × 10^−4^
w_1_ = 0.27, others unchanged	0.22	1.78	1.22	1.4 × 10^−4^
w_1_ = 0.33, others unchanged	0.20	1.82	1.18	1.1 × 10^−4^
w_2_ = 0.18, others unchanged	0.22	1.83	1.17	1.3 × 10^−4^
w_2_ = 0.22, others unchanged	0.20	1.77	1.23	1.2 × 10^−4^
w_3_ = 0.27, others unchanged	0.22	1.75	1.25	1.5 × 10^−4^
w_3_ = 0.33, others unchanged	0.20	1.85	1.15	1.1 × 10^−4^
w_4_ = 0.18, others unchanged	0.22	1.76	1.24	1.4 × 10^−4^
w_4_ = 0.22, others unchanged	0.20	1.84	1.16	1.2 × 10^−4^

**Table 2:** Quantitative correlation analysis between the theoretical optimized cost function and measured bit error rate. The primary dominant factors for each flow-rate regime were systematically identified through cross-spectral coherence analysis and time–frequency morphological evaluations of the dynamically acquired acoustic noise field.

Flow Rate (L/min)	Correlation (R^2^)	RMSE (Dimensionless)	MAPE (%)	Primary Dominant Factor
0–150	0.98	0.04	3.5	Elastic wave propagation
150–300	0.93	0.11	6.8	Fluid pulsation noise
300–400	0.86	0.24	13.2	Turbulence scattering

## Data Availability

The original contributions presented in this study are included in the article. Further inquiries can be directed to the corresponding authors.
